# Pediatric awake epilepsy surgery: Intraoperative language mapping utilizing digital video gaming and electrocorticography

**DOI:** 10.1016/j.ebr.2021.100521

**Published:** 2021-12-25

**Authors:** Faisal Alotaibi, Ali Mir, Mona Al-Faraidy, Tareq Jallul, Raidah Al-Baradie

**Affiliations:** a College of Medicine, Alfaisal University, Riyadh, Saudi Arabia; b Neuroscience Centre, King Faisal Specialist Hospital and Research Center, Riyadh, Saudi Arabia; c Neuroscience Centre, King Fahad Specialist Hospital, Dammam, Saudi Arabia; d Anesthesia Department, King Fahad Specialist Hospital, Dammam, Saudi Arabia

**Keywords:** ECOG, Electrocorticography, EEG, electroencephalography, MRI, magnetic resonance imaging, FDG-PET, Fluorodeoxyglucose-positron emission tomography, SPECT, Single-photon emission computed tomography, Awake, Children, Epilepsy, Language mapping, Electrical stimulation

## Abstract

Intraoperative functional language mapping is vital to minimize the risks associated with surgical removal of the seizure onset zone in selected patients with epilepsy. In children, this method has been reported extraoperatively by the placement of invasive electrodes to map the language area and monitor epileptic activity. It is difficult from a technical standpoint to perform an awake craniotomy and language mapping in young children under 10 years of age. Here we report a 9-year-old boy suffering from drug-resistant non-lesional epilepsy who underwent extraoperative and intraoperative electrical stimulation with successful identification of Broca’s language area. Electrocorticography (ECOG) was applied intraoperatively in a continuous manner utilizing grid electrodes before the skin opening. We found that the use of visual digital video games facilitated extraoperative and intraoperative cortical mapping. Cortical language inhibition by electrical stimulation was elicited at an amplitude of 7 mA (100 μs single-phase duration and 50 Hz pulse width). Resection of the seizure onset zone was completed safely. The post-resection ECOG revealed the disappearance of epileptogenic electrographic discharges at the seizure onset contacts and at other involved contacts in the epileptogenic zone. After surgery, the child recovered well with no language deficits and remained seizure-free. The child remembered only the video game test that was performed intraoperatively. This report highlights safety strategies for awake language mapping in pediatrics and the importance of the perioperative use of a visual digital video game and continuous ECOG, in addition to the use of targeted language cortex stimulation to facilitate faster and safer intraoperative language mapping under awake conditions in this age group.

## Highlights

• Awake craniotomy for language mapping in children requires a specific perioperative strategy.
• Extraoperative language cortical mapping using a grid electrode provides more optimal setting that reduces anxiety and improves compliance.
• The utilization of video game familiar to the pediatric patient reduce anxiety and enhance cooperation during awake language cortical mapping.

## Introduction

Surgical intervention is an effective treatment for drug-resistant epilepsy in children [1]. Intraoperative electrical stimulation for language cortex mapping is often performed in adult and adolescent patients, though rarely in young children [2]. Subsequent to the first awake craniotomy for epilepsy performed by Vector Horsley in late 1800, Penfield and Jasper described their extensive experience with cortical mapping under awake conditions in adolescents and adults [3], [4]. Penfield and Jasper utilized intraoperative electrocorticography (ECOG) in children as young as 4 years of age. There are several reports of awake craniotomy in children; however, this technique is primarily employed in tumor cases [5], [6], [7]. Preoperative mapping using noninvasive techniques such as functional magnetic resonance imaging (fMRI) is beneficial in many patients with epilepsy originating at, or in the vicinity of, eloquent cortex, including the motor or language cortices. In young children, fMRI can provide a hemispheric lateralization and language regional localization [8]. In certain reports, fMRI was found to be less specific in the precise anatomical localization as compared to electrical cortical stimulation [9], [10]. Extraoperative cortical stimulation using invasive implanted electrodes is considered to be an effective method to map language areas prior to epilepsy surgery in young children [11]. However, grid electrodes may move during the surgical exposure to remove the epileptic focus. Thus, the most precise method of identifying the language area is intraoperative cortical stimulation under awake conditions [5], [12]. Awake craniotomy for cortical language mapping is very challenging in younger children and requires a specific perioperative technique to ensure a successful cortical mapping process [13], [14]. This case report describes an awake craniotomy technique to map Broca’s language area by intraoperative cortical stimulation, with the patient aided by interactive video games to reduce anxiety and enhance engagement with the given intraoperative task. Another feature of this technique involved the continuous application of the ECOG to establish electrographical landmarks of the grid contacts as a means of providing additional information to the anatomical landmarks.

## Case study methodology

After the approval of the research institutional review board, we report a 9-year-old boy who was diagnosed with drug-resistant epilepsy at the age of 7. The patient presented with two semiologies. The first was focal-aware motor seizures characterized by right upper-limb extension and aphasia, in addition to versive head movement to the right side and unilateral grimacing with and without focal to bilateral tonic-clonic seizures. The second was bilateral tonic-clonic seizures that occurred during wakefulness and sleep. The overall average seizure frequency was two every week. His epilepsy was uncontrolled despite trials of multiple antiseizure medications. He had no history of epilepsy risk factors and a normal neurological examination with no dermatological stigmata.

### Presurgical evaluation

**Fig. 1:**
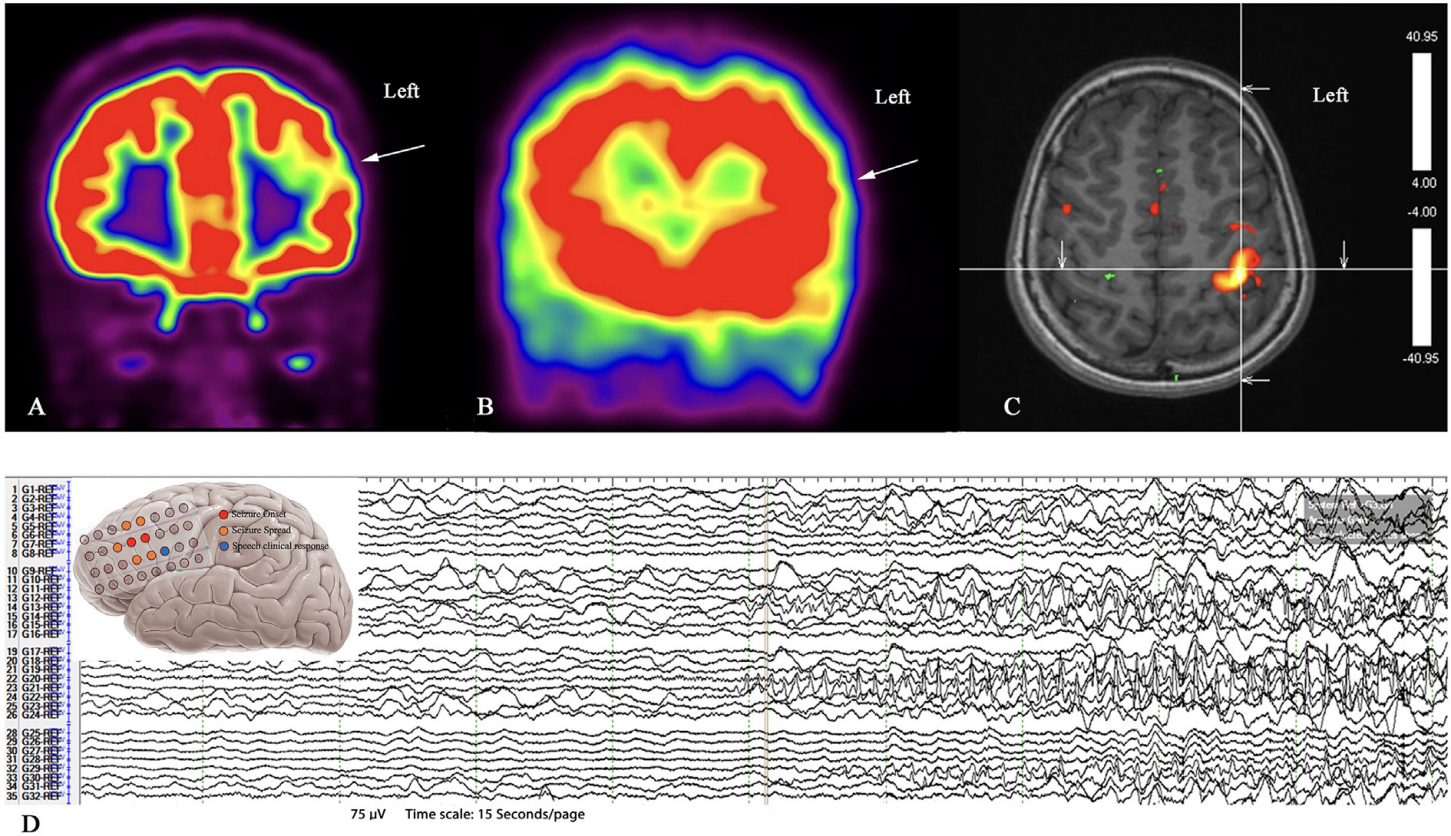
Functional neuroimages and invasive EEG seizure onset zone. A) 18 FDG-PET scan brain coronal view showing a focal hypometabolism at the left frontal area. B) Ictal SPECT coronal view showing a slight hyperperfusion at the left frontal region. C) Motor hand cortex area fMRI. Note that language could not be tested by fMRI. D) Invasive EEG ictal onset featuring the localized seizure onset zone.

Video-electroencephalography (EEG) monitoring revealed rare interictal sharp waves over the left frontocentral and left anterior frontal regions evident during wakefulness and sleeping. Ictal EEG showed a left hemispheric seizure onset in the frontocentral region. Brain magnetic resonant imaging (MRI) at 3 T showed no structural abnormalities. Fluorodeoxyglucose-positron emission tomography (FDG-PET) scan revealed focal hypometabolism at the left dorsolateral anterior frontal region. Interictal single-photon emission computed tomography (SPECT) revealed hypoperfusion at the left anterior lateral frontal region. Ictal SPECT showed hyperperfusion at the left lateral frontal and, to a certain extent, at the mesial anterior frontal regions Fig. 1A &B. Neuropsychological evaluation of the patient revealed average IQ and normal-for-age working memory.

### Invasive EEG monitoring and extraoperative language mapping

Subdural grid and strip electrodes were placed to cover the left frontal pole and dorsolateral and mesial frontal lobe. The temporal, orbitofrontal, and parietal lobes were covered as well. A grid with 32 contacts, 4.5 mm in diameter, and spaced 10 mm apart (PMT®; Chanhassen, MN, USA) was placed over the patient’s dorsolateral frontal region, where it encompassed the expressive speech area of the motor cortex for additional eloquent cortical stimulation mapping.

Seizure onset was identified electrographically at the left middle frontal gyrus, with activities beginning at least 3 s prior to speech arrest and other seizure semiology. The seizure onset was localized at two electrodes on the grid, as is shown in Fig. 1D. Extraoperative electrical cortical stimulation (Nihon Kohden® MS120, Foothill Ranch, CA, USA) was performed utilizing the following parameters: 50 Hz frequency, 0.2 millisecond pulse, up to 5 s train, and 1–8 mA current intensity. Stimulus intensity was increased in a stepwise manner starting at 1 mA until a clinical response was achieved or after discharge was obtained. A video game based on number counts and word generation utilizing an iPad screen (Apple Inc., USA) was provided to the patient before and during cortical stimulation. The video game consisted of numbers appearing on the screen, which the patient needed to identify quickly to reach a certain sequence that would then be followed by a reward score announced on the screen. The patient was also given a picture-naming video game that followed a pattern similar to that of the number game. The child was familiarized with the game prior to the cortical stimulation. Speech hesitation was elicited using 6 mA stimulation intensity over the inferior frontal gyrus corresponding to the anatomical Broca’s area; however, no classic speech arrest was confirmed Fig. 1D. A habitual focal aware seizure was induced by stimulating the contact located near the seizure onset using 6 mA stimulation intensity. This limited further extraoperative language mapping.

### Awake craniotomy with continuous ECOG monitoring

The patient was brought to the operating room accompanied by his father while engaged in the video game on the iPad screen. This method has been found to be effective in reducing preoperative anxiety in children by our anesthesia team. The patient was sedated with remifentanil and propofol at an infusion rate of 0.06 μg/kg/minute and 60/kg μg/kg/minute, respectively. This was stopped before the awake cortical mapping procedure began.

**Fig. 2:**
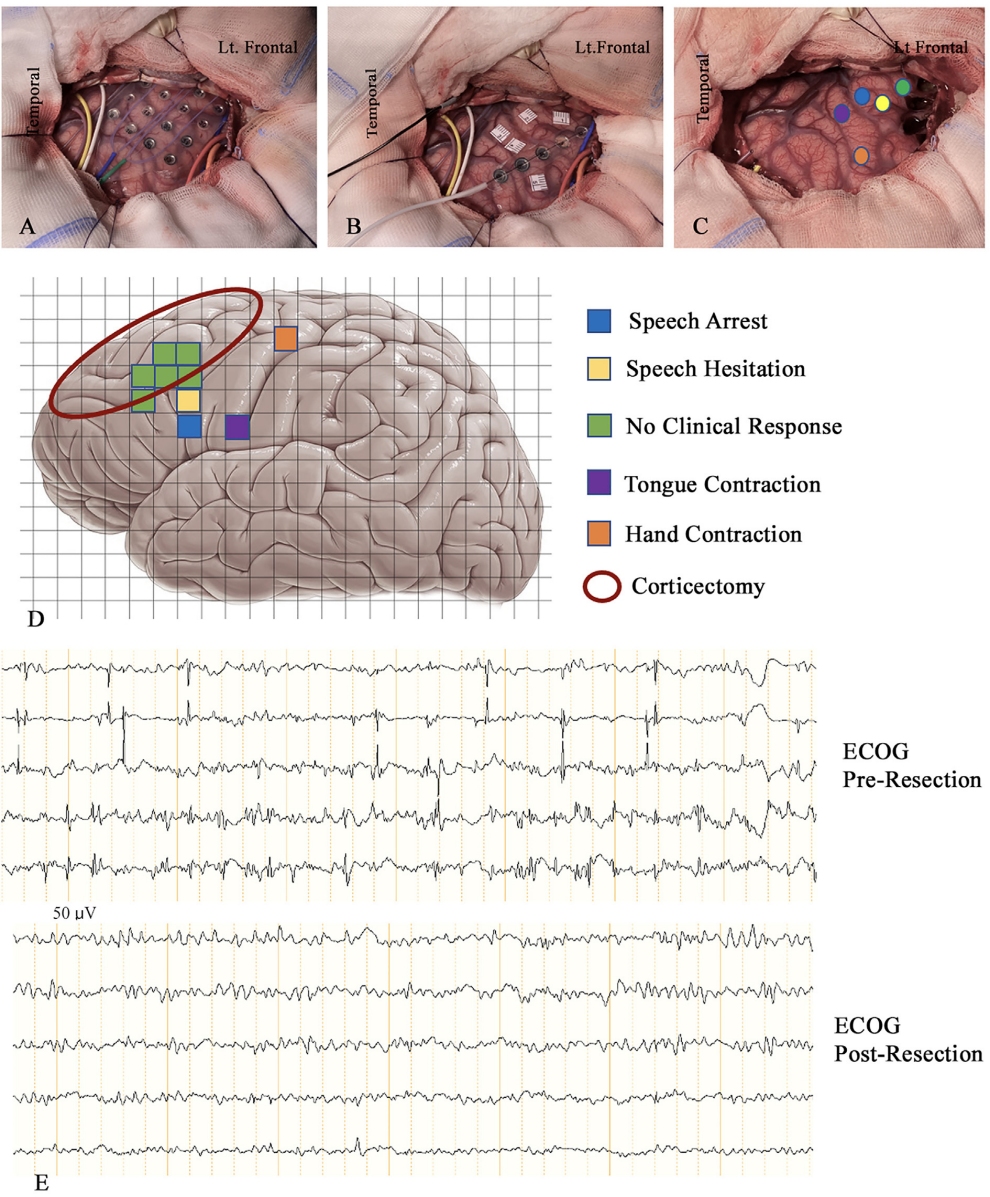
Results of language cortical mapping and electrocorticography (ECOG). A) Cortical exposure with the grid in place. B) Cortical mapping positive area after removing the grid and using a strip electrode for ECOG. C & D) Cortical language and motor mapping positive and negative clinical responses labeled in different colors in correlation to the corticectomy area. E) Pre- and post-resection ECOG.

The head was placed over a horseshoe head holder exposing the left side of the head Fig. 2A-C. Before beginning the procedure, the invasive electrode cables were connected to ECOG for perioperative recording. The maximal cluster of interictal spikes matched the location of the spikes during extraoperative recording and did not change during the cortical exposure phase of the surgical procedure. This indicated that the location of the grid electrodes did not change during surgical exposure. We applied a transparent operative drape used specifically for awake craniotomies.

### Intraoperative cortical stimulation for language mapping

After removing the grid electrodes and labeling the cortical location of the seizure onset and the preoperatively identified Broca’s area, the patient was brought out of sedation and the iPad was reintroduced to him with the same video games used during the extraoperative cortical mapping. Once the child was engaged in the video game, electrical stimulation was initiated applying Penfield’s technique using the Ojemann stimulator (Integra OCS2), with a 5 mm distance between 1 mm contact electrodes. Electrical stimulation was applied with a constant current generator producing a square wave train with biphasic pulses of 2 ms duration at a frequency of 50 Hz. The stimulus was initially applied at 1 mA and was gradually increased by 0.5–1 mA, and the duration on the cortical surface was 2–4 s for each stimulation. Cold saline was prepared in case of intraoperative seizure. The stimulation targeted the preoperatively located speech hesitation area for a faster mapping process. Speech arrest was elicited at 7 mA. The stimulation site was then moved 20 mm away from Broca’s area to define the cortical speech area, then moved 10 mm closer to Broca’s area. Speech hesitation (paraphasia) occurred at 7 mA at the site 10 mm from Broca’s area, with no afterdischarges on ECOG or intraoperative seizure. The tongue cortical area was identified at 5 mA posterior to the speech area Fig. 2D & E. Resection of the seizure onset zone was carried out safely using an ultrasonic surgical aspirator, removing all involved cortical areas. The child recovered post-procedure with no speech dysfunction or any neurological deficits. He remained seizure-free at 6-month follow-up and at the time of writing this report.

## Discussion

Awake craniotomy is widely used in adult and adolescent patients to map language and motor areas; similar methods are rarely performed in young children [2], [3], [6], [15]. We are reporting on this case, illustrating the methodology applied. On the other hand, it is considered the first awake language mapping in a young child reported from our country to our knowledge. In young children, the application of extraoperative language cortical mapping using a grid electrode is appropriate due to the presence of family, which provides a more optimal setting that reduces anxiety and improves compliance and cooperation. This allows for effective cortical stimulation test conduction with high reliability. In addition, this setting allows children as young as 2 years old to be candidates for this procedure [16]. Schevon and colleagues showed that the rate of extraoperative identification of a language area in children below 10 years of age is lower than in older children and adults [17]. Although extraoperative stimulation is important for delineating the language area in relation to the seizure onset zone, the grid electrodes may move during the exposure phase of the definitive epilepsy surgery. We used the continues ECOG method to ensure that the grid remained in place and did not shift, using the electrographical spikes as markers for certain contacts. However, precise intraoperative localization of the language cortex is thus critical during resection of the seizure onset zone in the vicinity of this eloquent area [18].

In this report, a video game familiar to the patient was utilized to reduce anxiety and increase compliance and cooperation during awake language cortical mapping. The same technique was used in an extraoperative setting and resulted in the effective engagement of the child during cortical electrical stimulation without noticeable discomfort caused by the operating room environment. It was difficult to run a psychometric assessment to quantify the anxiety level for this child during such procedure. The awake craniotomy condition that we employed is similar to what has been utilized over many decades and reported in the literature [6], [11], [15], [18]. Continuous ECOG during the exposure allowed for monitoring of any changes in the grid location by tracing the spikes at each individual contact located at the seizure onset. Furthermore, ECOG enabled the verification of electrical stimulation artifacts and afterdischarges during cortical mapping. The bipolar cortical electrical stimulation method permits a localized stimulation field with an effect area of 1.2 mm^2^ that minimizes current spread and lowers the chance of intraoperative seizure [11]. In our case, Broca’s area boundaries were mapped using a bipolar probe with no adverse events. The use of an iPad projecting a familiar video game visualizing numbers and pictures for verb generation facilitated the conduction of cortical mapping.

Specialized cortical area for language has been identified in the cortex prenatally, suggested by the asymmetry of anatomical language cortices in both hemispheres [19]. Language transformation has been seen after hemispheric disconnection surgery, however, reflecting the degree of language cortical plasticity during early childhood brain development [20], [21]. During tumor surgeries, Sanai and colleagues identified different language-associated cortical areas around the primary expressive speech area [6]. In our patient, Broca’s area was identified at the opercularis cortical area, which was the expected anatomical location, indicating no alteration in this cortical function location by the epileptogenic zone. In general, language area connectivity is complex, and despite several decades of functional anatomy research, this area remains difficult to characterize [22]. Future direction with the advancement in different techniques and technologies of cortical functional mapping might change the current method of cortical mapping during epilepsy surgery in children. These include diffusion tensor images, cortico-cortical evoked potentials, and ECOG high-gamma modulation language mapping techniques [23], [24], [25]. This will indeed enhance epilepsy surgery safety in children and adults during resection of seizure onset zone at an eloquent language cortical area.

## Conclusion

Awake craniotomy for language mapping in children requires a specific perioperative strategy. We described in this case report a quick, targeted method of language cortex intraoperative electrical stimulation using a video game to enhance patient engagement. Applying the ECOG method during surgical exposure provided electrographical landmarks for the grid contacts in addition to the anatomical landmarks. The future direction of language mapping in children may depend on perioperative techniques and technology such as diffusion tensor imaging, high-gamma modulation, and cortico-cortical evoked potentials.

## Ethical statement

All authors are compliant with all relevant ethical regulations. Study funded in part by the Saudi Epilepsy Society

## Contributors

All the authors were involved in the conception, design, and approval of the work. FA, AM, MA, and RA were involved in data collection, manuscript design, review, and approval.

## Declaration of Competing Interest

The authors declare that they have no known competing financial interests or personal relationships that could have appeared to influence the work reported in this paper.

## References

[1] Dwivedi R., Ramanujam B., Chandra P.S., Sapra S., Gulati S., Kalaivani M. (2017). Surgery for drug-resistant epilepsy in children. N Engl J Med.

[2] Lohkamp L.-N., Mottolese C., Szathmari A., Huguet L., Beuriat P.-A., Christofori I. (2019). Awake brain surgery in children-review of the literature and state-of-the-art. Childs Nerv Syst.

[3] Penfield W., Jasper H. (1954). Epilepsy and the functional anatomy of the human brain. South Med J.

[4] Horsley V. (1887). Remarks on ten consecutive cases of operations upon the brain and cranial cavity to illustrate the details and safety of the method employed. Br Med J.

[5] Gallentine W.B., Mikati M.A. (2009). Intraoperative electrocorticography and cortical stimulation in children. J Clin Neurophysiol.

[6] Sanai N., Mirzadeh Z., Berger M.S. (2008). Functional outcome after language mapping for glioma resection. N Engl J Med.

[7] Duffau H. (2015). Stimulation mapping of white matter tracts to study brain functional connectivity. Nat Rev Neurol.

[8] Szaflarski J.P., Holland S.K., Schmithorst V.J., Byars A.W. (2006). fMRI study of language lateralization in children and adults. Hum Brain Mapp.

[9] Pur D.R., Eagleson R., Lo M., Jurkiewicz M.T., Andrade A., de Ribaupierre S. (2021). Presurgical brain mapping of the language network in pediatric patients with epilepsy using resting-state fMRI. J Neurosurg Pediatr.

[10] Rolinski R., Austermuehle A., Wiggs E., Agrawal S., Sepeta L.N., Gaillard W.D. (2019). Functional MRI and direct cortical stimulation: Prediction of postoperative language decline. Epilepsia.

[11] Ojemann S.G., Berger M.S., Lettich E., Ojemann G.A. (2003). Localization of language function in children: results of electrical stimulation mapping. J Neurosurg.

[12] Gonen T., Gazit T., Korn A., Kirschner A., Perry D., Hendler T. (2017). Intra-operative multi-site stimulation: Expanding methodology for cortical brain mapping of language functions. PLoS ONE.

[13] Alcaraz García‐Tejedor G., Echániz G., Strantzas S., Jalloh I., Rutka J., Drake J. (2020). Feasibility of awake craniotomy in the pediatric population. Paediatr Anaesth.

[14] Balogun J.A., Khan O.H., Taylor M., Dirks P., Der T., Carter Snead III O. (2014). Carter Snead Iii O, Weiss S, Ochi A, Drake J, Rutka JT: Pediatric awake craniotomy and intra-operative stimulation mapping. J Clin Neurosci.

[15] Delion M., Terminassian A., Lehousse T., Aubin G., Malka J., N'Guyen S. (2015). Specificities of awake craniotomy and brain mapping in children for resection of supratentorial tumors in the language area. World Neurosurg.

[16] Duchowny M., Jayakar P., Harvey A.S., Resnick T., Alvarez L., Dean P. (1996). Language cortex representation: effects of developmental versus acquired pathology. Ann Neurol.

[17] Schevon C.A., Carlson C., Zaroff C.M., Weiner H.J., Doyle W.K., Miles D. (2007). Pediatric language mapping: sensitivity of neurostimulation and Wada testing in epilepsy surgery. Epilepsia.

[18] Ojemann G, Ojemann J, Lettich E, Berger M: Cortical language localization in left, dominant hemisphere. An electrical stimulation mapping investigation in 117 patients. 1989. *J Neurosurg* 2008, 108(2):411-421. 10.3171/JNS/2008/108/2/0411 18240946

[19] DeVos K.J., Wyllie E., Geckler C., Kotagal P., Comair Y. (1995). Language dominance in patients with early childhood tumors near left hemisphere language areas. Neurology.

[20] Alotaibi F., Albaradie R., Almubarak S., Baeesa S., Steven D.A., Girvin J.P. (2021). Hemispherotomy for epilepsy: the procedure evolution and outcome. Can J Neurol Sci.

[21] Corina D.P., Loudermilk B.C., Detwiler L., Martin R.F., Brinkley J.F., Ojemann G. (2010). Analysis of naming errors during cortical stimulation mapping: implications for models of language representation. Brain Lang.

[22] Hickok G., Poeppel D. (2007). The cortical organization of speech processing. Nat Rev Neurosci.

[23] Arya R., Horn P.S., Crone N.E. (2018). ECoG high-gamma modulation versus electrical stimulation for presurgical language mapping. Epilepsy Behav.

[24] Jeong J.-W., Asano E., Juhász C., Chugani H.T. (2015). Localization of specific language pathways using diffusion-weighted imaging tractography for presurgical planning of children with intractable epilepsy. Epilepsia.

[25] Yamao Y., Matsumoto R., Kikuchi T., Yoshida K., Kunieda T., Miyamoto S. (2021). Intraoperative brain mapping by cortico-cortical evoked potential. Front Hum Neurosci.

